# Cellular RNA Binding Proteins NS1-BP and hnRNP K Regulate Influenza A Virus RNA Splicing

**DOI:** 10.1371/journal.ppat.1003460

**Published:** 2013-06-27

**Authors:** Pei-Ling Tsai, Ni-Ting Chiou, Sharon Kuss, Adolfo García-Sastre, Kristen W. Lynch, Beatriz M. A. Fontoura

**Affiliations:** 1 Department of Cell Biology, University of Texas Southwestern Medical Center, Dallas, Texas, United States of America; 2 Department of Biochemistry and Biophysics, University of Pennsylvania School of Medicine, Philadelphia, Pennsylvania, United States of America; 3 Departments of Microbiology, Medicine, Infectious Diseases, Global Health and Emerging Pathogens Institute, Mount Sinai School of Medicine, New York, New York, United States of America; Johns Hopkins University - Bloomberg School of Public Health, United States of America

## Abstract

Influenza A virus is a major human pathogen with a genome comprised of eight single-strand, negative-sense, RNA segments. Two viral RNA segments, NS1 and M, undergo alternative splicing and yield several proteins including NS1, NS2, M1 and M2 proteins. However, the mechanisms or players involved in splicing of these viral RNA segments have not been fully studied. Here, by investigating the interacting partners and function of the cellular protein NS1-binding protein (NS1-BP), we revealed novel players in the splicing of the M1 segment. Using a proteomics approach, we identified a complex of RNA binding proteins containing NS1-BP and heterogeneous nuclear ribonucleoproteins (hnRNPs), among which are hnRNPs involved in host pre-mRNA splicing. We found that low levels of NS1-BP specifically impaired proper alternative splicing of the viral M1 mRNA segment to yield the M2 mRNA without affecting splicing of mRNA_3_, M4, or the NS mRNA segments. Further biochemical analysis by formaldehyde and UV cross-linking demonstrated that NS1-BP did not interact directly with viral M1 mRNA but its interacting partners, hnRNPs A1, K, L, and M, directly bound M1 mRNA. Among these hnRNPs, we identified hnRNP K as a major mediator of M1 mRNA splicing. The M1 mRNA segment generates the matrix protein M1 and the M2 ion channel, which are essential proteins involved in viral trafficking, release into the cytoplasm, and budding. Thus, reduction of NS1-BP and/or hnRNP K levels altered M2/M1 mRNA and protein ratios, decreasing M2 levels and inhibiting virus replication. Thus, NS1-BP-hnRNPK complex is a key mediator of influenza A virus gene expression.

## Author Summary

Influenza A virus is a major human pathogen, which causes approximately 500,000 deaths/year worldwide. In pandemic years, influenza infection can lead to even higher mortality rates, as in 1918, when ∼30–50 million deaths occurred worldwide. In this manuscript, we identified a novel function for the cellular protein termed NS1-BP as a regulator of the influenza A virus life cycle. We found that NS1-BP, together with other host factors, mediates the expression of a key viral protein termed M2. NS1-BP and its interacting partner hnRNP K specifically regulate alternative splicing of the viral M1 mRNA segment, which generates the M2 mRNA that is translated into the essential viral M2 protein. The M2 protein is key for viral uncoating and entry into the host cell cytoplasm. Altogether, inhibition of NS1-BP and hnRNP K functions regulate influenza A virus gene expression and replication. In sum, these studies revealed new functions for the cellular proteins NS1-BP and hnRNP K during viral RNA expression, which facilitate the influenza A virus life cycle.

## Introduction

Influenza A virus belongs to the *Orthomyxoviridae* family of RNA viruses and infects mammals and birds. Pathogenic strains of influenza A virus cause high mortality in humans, which usually results in ∼250,000 to 500,000 deaths/year worldwide [1]. In pandemic years, influenza infection can lead to even higher mortality rates, as in 1918, when at least 20 million deaths occurred worldwide [2]. Influenza A virus is an enveloped virus with a genome comprised of eight single-strand, negative-sense RNA segments that encode an increasing list of proteins [3]. Projecting from the viral surface are two glycoproteins, hemagglutinin (HA) and neuraminidase (NA), which determine the subtypes of influenza A viruses. Underneath the lipid bilayer, there are viral ribonucleoprotein complexes (vRNPs) comprised of RNAs and viral polymerase complex (PB1, PB2, and PA). Each viral RNA segment is wrapped with viral nucleoprotein (NP), and the viral polymerase complex binds to a panhandle/fork/corkscrew structure formed by the complementary base pairing at the 5′ and 3′ ends of the untranslated regions of viral RNAs [4]. In addition, N40 is a newly identified viral protein derived from the PB1 segment whose function is unclear [5], and some viruses also encode the PB1-F2 protein, which is expressed from a different open reading frame within the PB1 segment [6], [7], [8]. Recently, a ribosomal frameshift product derived from the RNA segment that encodes PA has been identified and termed PA-X [9]. This protein was then shown to modulate host response [9]. Furthermore, additional products from the PA segment have been reported including PA-N155 and PA-N182, which may also function in virus replication [10].

Two of the influenza virus RNA segments generate spliced products: NS segment encodes the non-structural protein (NS1) and nuclear export protein (NEP/NS2); M segment encodes the matrix protein (M1) and ion channel (M2). More recently, a splicing variant of M2 has been identified [11]. In addition, another splice product of the NS segment termed NS3 has been identified by adaptation of a human virus in a mouse host but its function remains unknown [12].

Influenza A virus initiates infection by attaching to sialic acids on the host cell surface via its HA protein, and then enters the cell through endocytosis. The low pH environment of the endosome triggers membrane fusion between the viral envelope and endosomal membrane, which is induced by HA. The viral core is acidified by the opening of the M2 ion channel, resulting in the release of the viral genome into the cytoplasm. Influenza virus replication depends on cellular factors present in the nucleus, so the released cytoplasmic vRNPs are imported into the nucleus and generate two positive-sense RNAs, mRNA and cRNA. Viral mRNA is then exported to the cytoplasm for translation. On the other hand, cRNP is used as template for producing progeny vRNA. The newly synthesized vRNPs are exported to the cytoplasm where new viral particles are assembled and then exit the host cell by budding [13].

The influenza A virus non-structural protein (NS1) down-regulates host pathways to evade the host innate immune system. In the cytoplasm, NS1 represses interferon α/β production by inhibiting signaling pathways [14]. In the nucleus, NS1 interacts with constituents of the mRNA processing and export machineries, including CPSF and poly(A) binding protein II [15], and the mRNA export complex containing the NXF1/TAP-p15/NXT export receptors [16]. These nuclear interactions result in blockage of host mRNA nuclear export [16], [17], which requires pyrimidines and prevents expression of mRNAs that encode antiviral factors [17]. In addition, NS1 was found to interact with a host protein termed NS1 binding protein (NS1-BP), which was previously shown to inhibit pre-mRNA splicing of a reporter gene *in vitro* [18]. Upon infection, NS1-BP was dispersed from speckles and re-distributed throughout the nucleus [18]. In this regard, NS1 was also found to alter the subcellular localization of splicing factors [19]. However, the role of NS1-BP during influenza A virus infection remained unknown. Here, we used biochemical and functional approaches to investigate NS1-BP function in the context of host cell-viral interaction. We found that NS1-BP interacted with the heterogenous nuclear ribonucleoprotein (hnRNP) K to promote splicing of M1 mRNA, which yields the viral M2 mRNA segment. Thus, NS1-BP and hnRNP K were revealed as key mediators of influenza A viral gene expression and replication.

## Results

### NS1-BP interacts with RNA binding proteins

**Figure 1:**
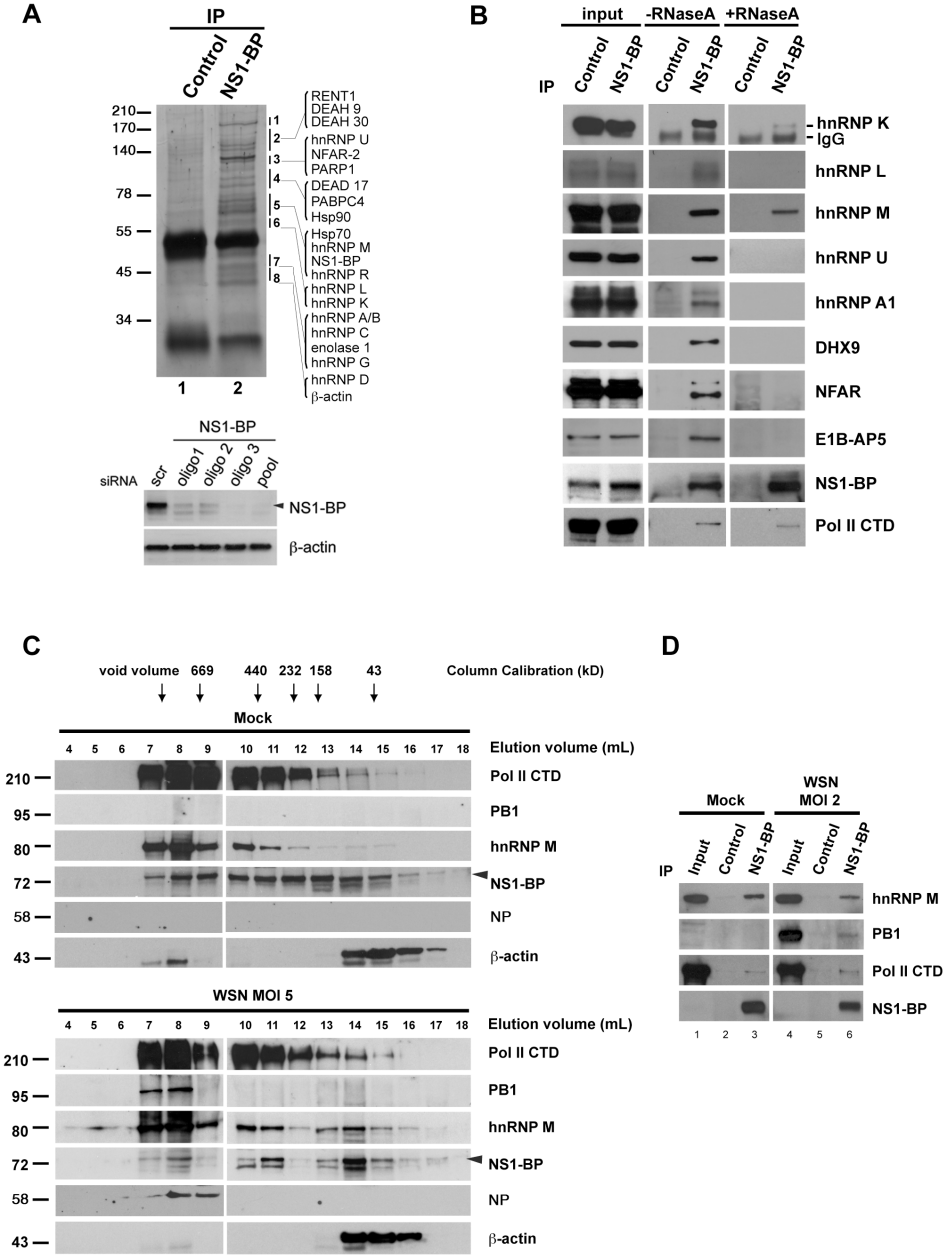
NS1-BP interacts with RNA-binding proteins, RNA polymerase II, and influenza A virus polymerase. (A) Top panel, HeLa cell lysates were immunoprecipitated with control IgG or NS1-BP antibodies. Interacting proteins were resolved by SDS-PAGE and identified by mass spectrometry. Bottom panel, A549 cells were treated with non-targeting or NS1-BP siRNAs (1, 2, and 3) for 48 h. Cell extracts were subjected to immunoblot analysis, which shows NS1-BP knockdown. β-actin served as loading control. (B) Immunoprecipitation was performed with control IgG or anti-NS1-BP antibodies, in the absence or presence of RNase A. Western blots were then performed with the depicted antibodies, selected based on the proteins identified in A. (C) A549 cells were mock-infected or infected with influenza virus at MOI 5 for 5 h. Cells were lysed and subjected to size exclusion chromatography. The fractions were concentrated by TCA precipitation and analyzed by western blot with the indicated antibodies. (D) A549 cells were mock-infected or infected with influenza virus (A/WSN/33) at MOI 2 for 16 h. Cell lysates were immunoprecipitated with control IgG or anti-NS1-BP antibodies. Western blots were performed with the depicted antibodies.

To investigate the function of NS1-BP, we first took a systematic biochemical approach to identify its interacting partners. We developed polyclonal antibodies that specifically recognized NS1-BP and immunoprecipitated NS1-BP from cell extracts (Figure 1A, top panel). Knockdown of NS1-BP with siRNA oligos followed by immunoblot analysis further demonstrate antibody specificity (Figure 1A, bottom panel). As also shown in Figure 1A and Table S1, we identified NS1-BP interacting partners by mass spectrometry, which included RNA-binding proteins. These proteins can be classified into the following categories: heterogeneous nuclear ribonucleoprotein and splicing factors (hnRNP family), RNA helicase (DEAD box polypeptide family), antiviral proteins [nuclear factor associated with dsRNA (NFAR-2), zinc finger antiviral protein], poly(A) binding protein, nuclear cap binding protein, chaperones (Hsp70 and Hsp90), and DNA damage repair protein [poly (ADP-ribose) polymerase, PARP]. Next, we examined whether these interactions were RNA-dependent or independent. NS1-BP was then immunoprecipitated in the absence or presence of RNase A. While NS1-BP interacted with RNA-binding proteins in the absence of RNase, most of these interactions were lost in the presence of RNase A. However, hnRNPs K and M remained associated with NS1-BP to a lesser extent upon RNase treatment (Figure 1B). These results suggested that NS1-BP is likely an RNP-binding protein, which could be involved in gene expression.

By comparing our proteomics results with other interactomes, we noticed that certain proteins that bound NS1-BP were found associated with the influenza A virus ribonucleoprotein (vRNP) or the heterotrimeric polymerase complex including PARP-1, NFAR-2, poly(A)-binding protein, hnRNP M, hnRNP A1, DDX17, Hsp70, and Hsp90 [20], [21], [22]. In addition, host-influenza polymerase interactomes also identified the largest subunit of cellular RNA polymerase II (Pol II CTD), which has been shown to bind the viral polymerase complex [23], [24]. Since NS1-BP shared some of the binding partners of the host-influenza polymerase interactome, we investigated the sedimentation profile of hnRNP M, viral and host polymerases, viral NP protein, which binds viral polymerase [25], and NS1-BP. Cell extracts were subjected to gel filtration and fractions were processed for immunoblot analysis with antibodies against NS1-BP, hnRNP M, PB1, NP, and host RNA polymerase II. We showed that a pool of NS1-BP, hnRNP M, PB1, NP, and a pool of the host polymerase II sedimented with high molecular weight complexes (Figure 1C). Interestingly, PB1 and NP are only found co-sedimenting with high molecular weight complexes. We then tested whether NS1-BP binds the cellular and viral RNA polymerases. Indeed, as shown in figure 1D, immunoprecipitation of NS1-BP revealed its interaction with both viral and host RNA polymerase II. Taking together all the results above, these findings indicated that NS1-BP physically interacts with hnRNPs and viral and host polymerases to likely regulate gene expression.

### NS1-BP and its interacting partner hnRNP K regulate splicing of influenza A virus M1 mRNA

**Figure 2:**
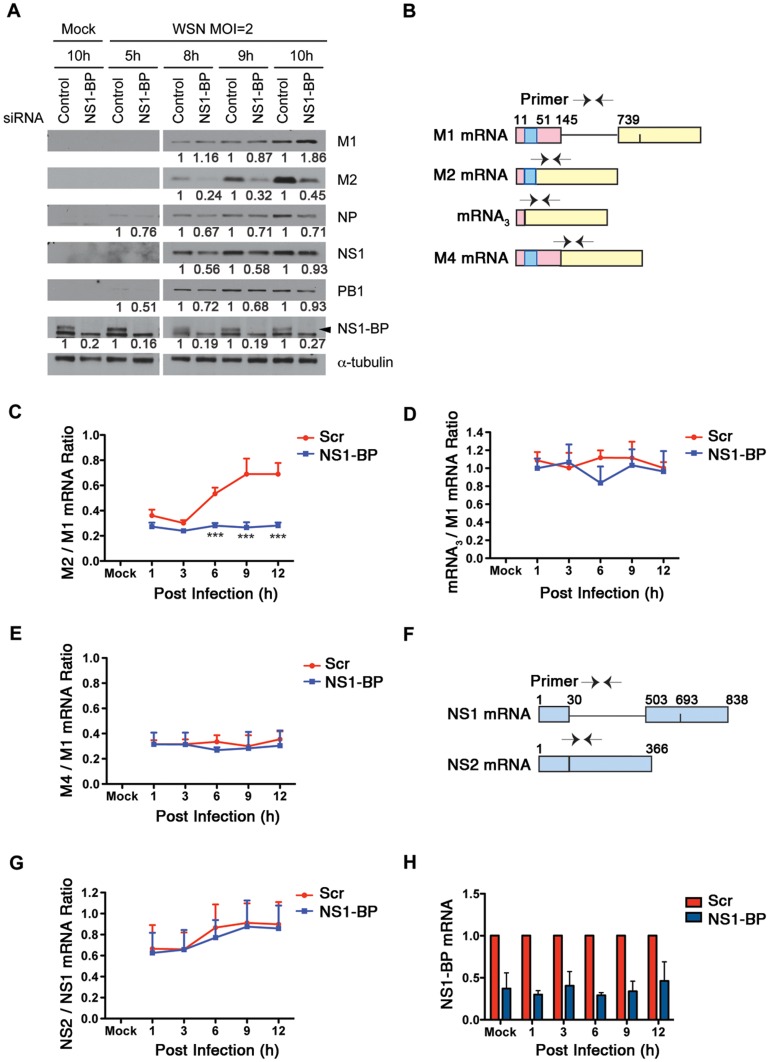
NS1-BP promotes splicing of influenza A virus M1 mRNA segment to yield proper levels of M2 mRNA and protein. (A) A549 cells were transfected with non-targeting or NS1-BP siRNAs for 48 h and infected with A/WSN/33 at MOI 2 for the indicated time points. Cells lysates were subjected to western blot analysis using antibodies against influenza virus proteins. α-tubulin was used as loading control. Each protein band was quantified by ImageJ and normalized to α-tubulin levels. (B) M1 mRNA and its alternatively spliced products are depicted and the arrowheads show primer positions for detection of various mRNAs in the following experiments. (C, D, and E) Cells were transfected and infected as in A and total RNA was isolated and analyzed by real-time RT-PCR with primers specific to M1, M2, mRNA_3_, and M4 mRNAs. Ratios of M2 mRNA to M1 (C) or to mRNA_3_ (D), or to M4 (E) over time are presented. (F and G) RNA samples from C were analyzed with primers specific to NS2 and NS1 viral mRNAs. (G) Ratios of NS2 mRNA to NS1 mRNA over time are shown. (H) NS1-BP mRNA levels were measured by real time RT-PCR in samples from C. Error Bars represent mean ± SD (n = 3).

To investigate the function of NS1-BP in the influenza virus life cycle, we knocked down NS1-BP in cells and infected them with A/WSN/33. We first assessed whether NS1-BP depletion affected viral entry or vRNP nuclear import by examining the localization of viral nucleoprotein (NP) in NS1-BP-depleted cells after infection and in the presence of the translation inhibitor cycloheximide. Compared to control, NS1-BP knockdown did not decrease nuclear NP staining (Figure S1), indicating that reduction of viral RNAs in NS1-BP depleted cells did not alter viral entry or vRNP nuclear import. Then, viral protein levels were assessed over time by immunoblot analysis (Figure 2A). While the levels of most viral proteins were not significantly altered, we observed an abnormal M2/M1 protein ratio in NS1-BP depleted cells suggesting a role for NS1-BP in regulating splicing of a specific viral mRNA, the M1 mRNA segment.

To investigate this potential effect on splicing, mRNA levels of M2 and M1 were measured by RT-qPCR. Indeed, the ratio of the M2 over M1 mRNAs decreased in cells depleted of NS1-BP and infected with A/WSN/33, indicating a splicing defect (Figure 2B and 2C). The dynamics of M2 and M1 expression in control cells was similar to what has been previously reported [26]. Different dynamics of M2 and M1 expression has also been reported [27], which may be due to differences in cell types. M2 mRNA is derived from alternative splicing of the M1 mRNA segment. In addition, the M1 mRNA segment can generate mRNA_3_ and, in certain strains such as A/WSN/33, mRNA4. However, it is unknown whether mRNA_3_ encodes a functional peptide [28], [29], [30], [31]. Recently, mRNA4 was shown to express an isoform of the M2 ion channel [11]. The 5′ splice site that yields mRNA_3_ is strong and has to be inhibited by influenza A virus polymerase to allow usage of the downstream weak splice site that generates the M2 mRNA, which happens later in infection. Therefore, we also examined mRNA_3_ and M4 levels relative to M1 mRNA over time and found no difference between control and NS1-BP knockdown cells (Figure 2D and 2E). Together, these findings reveal that sequences surrounding the M2-specific 5′ splice site and/or factors bound to this region are required for NS1-BP activity on splicing. We have also tested another viral segment, NS, which undergoes alternative splicing and generates the NS1 and NS2 mRNAs. In this case, no differences in ratio were observed between control and NS1-BP depleted cells (Figure 2F and 2G). Upon NS1-BP knockdown under these conditions, NS1-BP mRNA levels remained down-regulated throughout these mRNA measurements as compared to control (Figure 2H). Thus, these findings show that NS1-BP specifically regulates splicing of the M1 mRNA segment.

**Figure 3:**
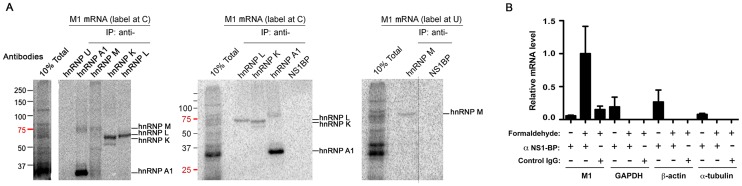
NS1-BP interacts with hnRNPs that directly bind M1 mRNA. (A) The entire M1 RNA was radiolabeled uniformly at either C (left) or U (right) residues, incubated with nuclear extract under splicing conditions, crosslinked with 254 nm light and digested with RNase. The reaction was then either resolved by SDS-PAGE (10% Total) or incubated in separate reactions with the antibodies indicated (IP: anti-). Immunoprecipitated proteins were resolved by SDS-PAGE. Position of molecular weight markers are also shown. Vertical dotted line shows that the samples were loaded in the same gel but not next to each other. Figure S4 shows immunoblot analysis of nuclear extract with antibodies against NS1-BP and hnRNP U, which did not directly bind M1 mRNA but are present in the extract. (B) A549 cells were infected with A/WSN/33 at MOI 5 for 5 h and were then cross-linked with 0.3% formaldehyde. Cells were lysed with RIPA buffer, and the lysates were immunoprecipitated with control IgG or NS1-BP antibodies. After a series of washes, the cross-link was reversed, and associated RNAs were isolated. Real-time quantitative RT-PCR was used to measure M1 mRNA. Housekeeping genes (GAPDH, β-actin, and α-tubulin) were included to test the specificity of the assay. Data was normalized to the viral M segment. The graph displays the mean ± SEM (n = 3). Comparison between cross-linked samples shows significant (p<0.05) differences between association of NS1-BP with M1 mRNA and the lack of association of NS1-BP with the 3 host mRNAs tested.

NS1-BP effect on M1 mRNA splicing is consistent with our findings of NS1-BP interaction with the influenza virus polymerase, which was previously reported to regulate splicing of the M1 mRNA [32]. In addition, our proteomics analysis showed NS1-BP interaction with hnRNPs previously known to be involved in splicing such as hnRNP M [33] and hnRNP K [34]. Thus, we tested whether NS1-BP and the hnRNPs that interacted with NS1-BP (Figure 1A and 1B) bound directly to M1 RNA. Capped and ^32^P-labeled M1 RNA was incubated with nuclear extracts and direct protein-RNA interactions were crosslinked with UV, followed by RNA digestion and immunoprecipitation with antibodies specific to various individual hnRNPs. The hnRNPs bound to a ^32^P-labeled nucleotide (C or U) were then detected. As shown in Figure 3A, hnRNP U and NS1-BP did not directly bind M1 RNA whereas hnRNPs A1, M, L, and K directly interacted with M1 RNA. By contrast, we detect no binding of hnRNP K to a control RNA derived from the CD45 pre-mRNA (Figure S2) [35], [36]. However, NS1-BP bound the viral M1 segment upon formaldehyde cross-linking (Figure 3B). In this case, A549 cells were infected with influenza A/WSN/33 virus at MOI 5 for 5 h, crosslinked with formaldehyde, and then lysed. Cell lysates were subjected to immunoprecipitation, and the associated viral RNAs were isolated and quantified by RT-qPCR. Each experiment included three conditions as following: immunoprecipitations were performed with NS1-BP antibodies using lysates with or without formaldehyde crosslinking, and control IgG was incubated with lysates subjected to formaldehyde crosslinking. The results indicate that formaldehyde cross-linked M1 mRNA to NS1-BP as opposed to three host mRNAs encoding GAPDH, β-actin, and α-tubulin, which were not cross-linked to M1 mRNA (Figure 3B). Since NS1-BP interacted with the M1 segment upon formaldehyde treatment but not upon UV crosslinking, these results indicate that NS1-BP likely requires adaptor proteins, such as hnRNPs, to interact with the M1 segment.

**Figure 4:**
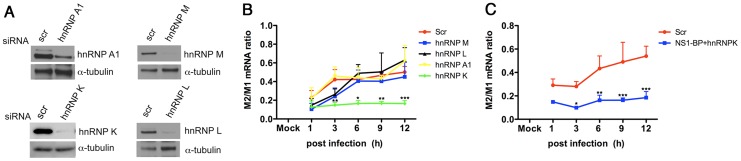
hnRNP K regulates M1 mRNA splicing. (A) Cell extracts from A549 cells transfected with non-targeting siRNAs or siRNAs that target various indicated hnRNPs for 48 h were subjected to immunoblot analysis with antibodies specific to each hnRNP. (B and C) A549 cells were transfected as in B and then infected with A/WSN/33 at MOI 2 for the indicated time points. In C, NS1-BP and hnRNP K were simultaneously knocked down. Total RNA was isolated at each depicted time point and analyzed by real-time RT-PCR with primers specific to M1 and M2 mRNAs. M2/M1 mRNA ratios are shown. Error bars represent mean ± SD (n = 3). *p<0.05, **p<0.01, ***p<0.001.

The hnRNPs that directly bound the M1 mRNA were then tested for regulation of M1 mRNA splicing in virus-infected cells. Each hnRNP was knocked down individually and the ratios of the M2 over M1 mRNAs were assessed over time. As shown in Figures 4A and 4B, only depletion of hnRNP K showed a striking decrease in M1 mRNA splicing, reducing the levels of M2 mRNA. Furthermore, simultaneous knockdown of NS1-BP and hnRNP K prevented M2 expression (Figure 4C), indicating that hnRNP K depletion was sufficient to inhibit M1 mRNA splicing. Taken together, a model could be proposed in which NS1-BP regulates M1 mRNA splicing via hnRNP K, resulting in M2 mRNA expression.

### NS1-BP and hnRNP K are required for optimal expression of viral proteins and viral replication

**Figure 5:**
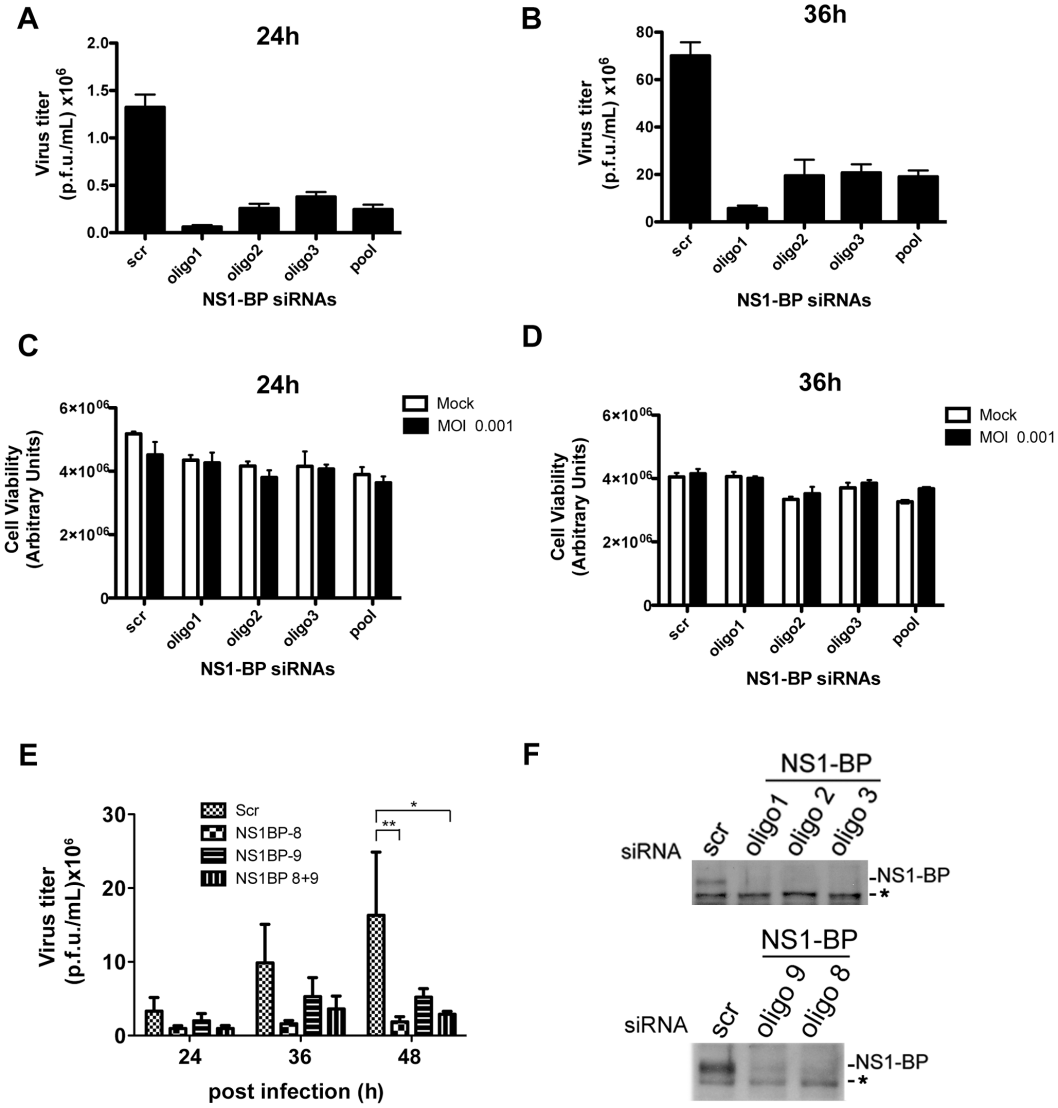
NS1-BP is required for optimal influenza A virus replication. (A) and (B) A549 cells were transfected with non-targeting or NS1-BP siRNAs for 48 h and infected with A/WSN/33 at MOI 0.001. Supernatants were harvested at 24 h and 36 h post-infection, and virus titer was determined by plaque assay. (C) and (D) After removal of supernatants from plaque assays in A and B, the remaining cells were used to measure ATP levels, at 24 h and 36 h post-infection, to determine cell viability. (E) Human bronchial epithelial cells (HBEC) were transfected and infected as in A except that different NS1-BP siRNAs were used. Supernatants were subjected to plaque assay. (F) Cell extracts from A549 cells transfected with siRNAs as in A and HBEC transfected with siRNAs as in E were subjected to immunoblot analysis with anti-NS1-BP antibodies. * denotes a cross-reacting band that serves as loading control. *p<0.05, **p<0.01.

To determine whether the effect of NS1-BP or hnRNP K on viral gene expression would result in alteration of viral protein levels and replication, we examined both parameters in cells depleted of NS1-BP or hnRNP K versus control cells. As shown in Figure 2A, the ratio of M2 to M1 at the protein level follows the effect of NS1-BP depletion on the ratio of M2 and M1 mRNAs (Figure 2C). Regulation of M2 expression by NS1-BP is an important step in the viral life cycle as M2 is the ion channel that allows entry of hydrogen ions from the endosome into the viral particle resulting in uncoating of the virus and release of viral content into the cytoplasm. Consequently, the observed effects of NS1-BP depletion on viral gene expression resulted in down-regulation of influenza A virus replication in A549 cells (Figure 5A and 5B). siRNA oligo 1 was more efficient in down-regulating virus replication (∼21 fold at 24 h and ∼12 fold at 36 h) than oligos 2 and 3 (∼4–6 fold). While all oligos down-regulated NS1-BP levels efficiently (Figure 5F), the range of viral titers is likely due to differences in the kinetics of knockdown for each siRNA oligo. ATP levels were also measured and the results did not show significant difference in cell death between control and NS1-BP depletion during the experimental period (Figure 5C and 5D), indicating that the decrease of virus replication in NS1-BP depleted cells was not due to cell death but likely due to NS1-BP effects on viral gene expression. We have also analyzed virus replication in human bronchial epithelial cells (HBEC) and observed a decrease in influenza A virus replication between ∼3–9 fold, depending on the siRNA oligo and depicted time points (Figure 5E and 5F).

**Figure 6:**
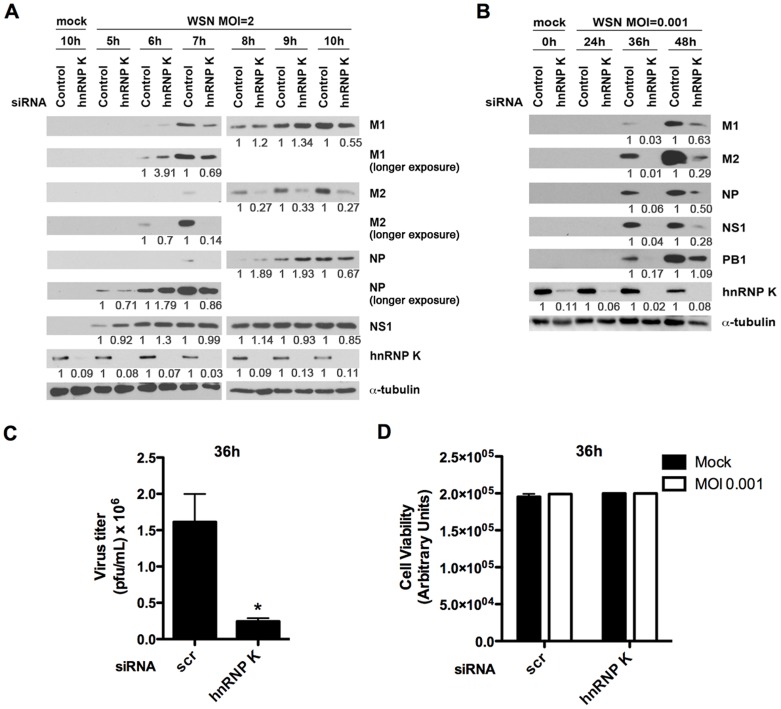
hnRNP K is required for optimal M2 protein production and influenza A virus replication. A549 cells were transfected with non-targeting or hnRNP K siRNAs for 48 h prior to infection. siRNA transfected cells were infected with A/WSN/33 at (A) MOI 2 or (B) MOI 0.001. Cells were harvested at the indicated hours post-infection, and viral protein accumulation was assessed by immunoblot analysis. Each protein band in (A) and (B) was quantified by ImageJ and normalized to α-tubulin levels. (C) Control or hnRNP K siRNA transfected cells were infected with A/WSN/33 at MOI 0.001. At 36 hours post-infection, cell supernatants were collected and subjected to viral titer analysis (n = 3) or (D) ATP level analysis to determine cell viability (n = 3, representative experiment). Error bars denote mean + SEM. *p<0.05.

Furthermore, depletion of hnRNP K followed by influenza virus infection at high MOI, single round of viral replication, resulted primarily in an abnormal ratio of M2 to M1 protein expression, which reduced M2 protein levels (Figure 6A) and demonstrated a dramatic consequence of the splicing defect shown in Figure 4B. Consequently, viral replication was clearly compromised upon hnRNP K depletion as infection at low MOI, in which multiple rounds of viral replication are allowed, showed diminished overall viral protein levels (Figure 6B) and viral titer (Figure 6C), without causing cell death (Figure 6D). In contrast, knockdown of hnRNPs A1, L, and M did not alter M2 levels but decreased M1 levels at 10 h post-infection (Figure S3). Collectively, these results demonstrate that NS1-BP and hnRNP K promote splicing of the M1 mRNA segment to produce the M2 mRNA, which encodes the M2 protein, an essential factor for viral replication.

## Discussion

In this study, we report a novel function for the cellular NS1-BP protein. NS1-BP interacted with cellular RNA-binding proteins, including splicing factors and RNA polymerase II, in the absence or presence of virus infection, suggesting a role in the regulation of gene expression. During infection, our results indicate that the influenza A virus RNA polymerase and viral RNAs associate with the NS1-BP/RNP complex to likely regulate viral RNA splicing of the M1 mRNA segment to generate the M2 protein. Furthermore, we identified hnRNP K as the interacting partner of NS1-BP that directly binds the M1 mRNA to promote splicing and induce expression of M2 mRNA. Thus, these findings revealed a novel function for NS1-BP and hnRNP K as key regulators of influenza A virus infection.

Influenza A virus polymerase transcribes and replicates the viral genome by interacting at the 5′ end with cRNA and vRNA. In addition to these primary roles, influenza A virus polymerase has been shown to regulate the choice of alternative 5′ splice sites in influenza A virus M1 mRNA [32]. The influenza A virus polymerase binds M1-specific sequences in M1 mRNA in addition to the 5′-terminal cap structure which results in the switch to the downstream M2 5′ splice site. Since NS1-BP binds the influenza A virus polymerase and together with hnRNP K regulates M1 mRNA splicing, a model can be proposed in which binding of the NS1-BP-hnRNP K complex to the influenza virus polymerase and M1 mRNA blocks the proximal 5′ splice site causing the switch to the downstream splicing site, yielding expression of M2 mRNA. In this regard, hnRNP K was previously shown to regulate splicing of HIV-1 pre-mRNA [34]. The acceptor site A7 on HIV-1 pre-mRNA has an essential role for expressing *tat* and *rev* mRNAs, which encode proteins essential for viral mRNA synthesis and export. hnRNP K was show to down-regulate A7 activity [34]. In addition, hnRNP K was reported as a repressor of hepatitis B virus replication [37] but promoted herpes simplex virus-1 propagation [38]. Together, these findings demonstrate that hnRNP K has a key regulatory role in the replication of evolutionarily diverse viruses and it can act in a positive or negative manner, depending on the context or virus.

Splicing of the M1 mRNA is a fundamental process for virus replication as it generates the M2 ion channel, which is essential for vRNP release into the host cytoplasm upon entry and for release of newly synthesized virions via budding from the plasma membrane [39]. Moreover, M2 expression is also required to prevent a premature conformational change of the HA of a subset of highly pathogenic H5 and H7 influenza A viruses [40]. Thus, depletion of NS1-BP or hnRNP K is likely to have more impact on these highly pathogenic avian influenza viruses.

The influenza A virus NS1 protein was recently shown to regulate the M1 mRNA splicing [27] and the involvement of the influenza A virus polymerase is not clear in this case. However, NS1 was previously shown to interact with the influenza A virus polymerase [41] and temperature-sensitive viruses expressing C-terminal truncations of NS1 presented defective RNA replication and late gene expression [42]. Therefore, future studies are necessary to determine whether NS1 regulates influenza A virus polymerase function in splicing and if this mechanism is related to NS1-BP function. One possibility is that influenza A virus may use NS1-dependent and independent mechanisms to regulate splicing of the M1 mRNA segment, which might occur at different stages of infection. In fact, host splicing can be differentially regulated depending on various stimuli, resulting in diverse effects such as differential protein-protein interactions, nucleo-cytoplasmic trafficking and transcription elongation [43]. Further experimentation using influenza A virus lacking the NS1 gene, which replicates in interferon-deficient systems [44], will be necessary to understand the impact of NS1 on NS1-BP function. Interestingly, NS1-BP levels did not affect splicing of the NS mRNA, indicating again differential splicing or factors involved in the splicing regulation of the M1 and NS1 mRNAs.

In sum, our findings reveal a novel role for NS1-BP and hnRNP K as host regulators of influenza A virus RNA biogenesis. In future studies, these findings may serve as paradigm to uncover a potential role for NS1-BP in splicing of specific classes of host pre-mRNAs. Since specific sequences surrounding the M2 5′ splice site are required for NS1-BP-hnRNP K activity on splicing, one can envisage NS1-BP regulating splicing, via hnRNP K, of a subset of host pre-mRNAs containing similar sequences. It would be interesting if host mRNAs regulated by NS1-BP and hnRNP K encode proteins involved in immune response during viral infection, which is an interesting topic for future investigation. In sum, the study presented here exposed novel host-viral interactions that regulate splicing of influenza A virus RNA, which play a significant role in the virus life cycle.

## Materials and Methods

### Cells and virus

Human lung adenocarcinoma epithelial cells (A549) were cultured in RPMI 1640 media supplemented with 10% heat-inactivated fetal bovine serum (Invitrogen), 100 units/mL penicillin, and 100 µg/mL streptomycin. HeLa and MDCK cells were cultured in DMEM media containing the same amount of serum and antibiotics described above. Human bronchial epithelial cells (HBEC) were cultured in Keratinocyte-SFM supplied with human recombinant epidermal growth factor, bovine pituitary extract (Invitrogen), 100 units/mL penicillin, and 100 µg/mL streptomycin. All cells were maintained at 37°C with 5% CO_2_.

Influenza virus (A/WSN/33) was propagated in MDCK cells. In brief, MDCK cells (2×10^7^) were seeded into a 15 cm dish. At the second day, cells were infected with viruses at multiplicity of infection (MOI) of 0.001 in 2.5 mL of infection media [EMEM, 10 mM HEPES, 0.2% BSA, 100 units/mL penicillin, 100 µg/mL streptomycin, and 0.5 µg/mL TPCK-treated trypsin]. After 1 h, cells were washed with PBS, overlaid with 10 mL infection media, and incubated until viral cytopathic effect was observed (∼36 h). Supernatants were harvested, centrifuged at 1000×g for 10 min, aliquotted, and stored at −80°C. Virus titer was determined by plaque assay (see below). Infection of A549 cells was performed in the same manner as above, except that the infection media did not contain TPCK-treated trypsin.

### Plasmids

Full-length cDNA clone of human NS1-BP (NM_006469.4) was purchased from OriGene Technologies, Inc. (Rockville, MD) and subcloned into *Sal* I and *Not* I sites of pGEX-4T-2 (GE Healthcare). The M2 was cloned from infected A549 cells by RT-PCR and inserted into the pGEM-T vector.

### Antibodies

GST-tagged full-length NS1-BP was used as antigen to develop polyclonal antibodies in rabbits. The specific antiserum was purified by affinity chromatography [45] with some modifications. In brief, GST and full-length GST-NS1-BP were expressed in BL21(DE3) cells and purified with glutathione-Sepharose 4B beads (GE Healthcare). Without elution, protein GST and GST-NS1-BP were covalently linked to glutathione-Sepharose beads by using the cross-linker dimethyl pimelimidate-HCl (Sigma). Crude serum was loaded onto the GST column to remove the non-specific antibodies and the flow through was collected and loaded onto the GST-NS1-BP column. After washes, the antibodies were eluted with 0.1 M Glycine (pH 2.5) and immediately neutralized to pH 7.5 by 3M Tris-HCl (pH 8.8). The eluted fractions were collected and concentrated in a Centricon (Millipore). The concentration of antibodies was adjusted to 1 µg/µL and used for western blotting at a dilution of 1∶1000.

hnRNP M monoclonal antibody (a gift from Dr. Maurice S. Swanson, University of Florida, FL) was used for western blot analysis at a 1∶4000 dilution. hnRNP A1 monoclonal antibody (a gift from Dr. Michael Matunis, Johns Hopkins School of Public Health, MD) was used at a dilution 1∶1000. NFAR polyclonal antibody (a gift from Dr. Glen Barber, University of Miami Miller School of Medicine, FL) was used at a dilution 1∶2000. hnRNP U monoclonal antibody (ImmuQuest) was used at a 1∶1000 dilution. hnRNPs K and L antibodies (abcam) were used at dilutions 1∶3000 and 1∶2000, respectively. E1B-AP5 polyclonal antibody (Proteintech Group Inc) was used at a 1∶2000 dilution. DHX9 monoclonal antibody (Abnova) was used at a 1∶500 dilution. RNA polymerase II monoclonal antibody, clone CTD4H8, recognized both phosphorylated and non-phosphorylated forms (Millipore), was used at a 1∶5000 dilution. Influenza A PB1 polyclonal antibody (Santa Cruz Biotechnology) was used at a 1∶5000 dilution. Anti-NS1 polyclonal antibody [46] was used at a 1∶30,000 dilution, and anti-M1/M2 monoclonal antibody [47] was used at a 1∶10,000 dilution. Anti-NP monoclonal antibody (ab20343, Abcam) was used at a 1∶3200 dilution for immunostaining. Anti-influenza A virion polyclonal antibodies (Meridian Life Science, Inc.) was used for NP western blot analysis at a 1∶10,000 dilution. β-actin monoclonal antibody (Sigma) was used at a 1∶20,000 dilution. α-tubulin monoclonal antibody (Sigma) was used at a 1∶20,000 dilution.

### Immunoprecipitation and mass spectrometry

HeLa cells were lysed with a buffer containing: 50 mM Tris, pH 7.5, 150 mM NaCl, 1% IGEPAL CA-630 (Sigma), 0.1 mM Na_3_VO_4_, 1 mM NaF, 1 mM DTT, 1 mM EDTA, 1 mM PMSF, 1× complete protease inhibitor cocktail (Roche), and 15% glycerol, for 20 min on ice. Cell lysates were centrifuged at 13,000×g for 15 min to pellet cellular debris. For experiments treated with RNase A, the enzyme was added to the lysates at a final concentration of 1 µg/mL. Five mg of cell lysates were pre-cleared with protein A beads (GE Healthcare) for 1 h; the pre-cleared lysates were incubated with new protein A beads and 6 µg of control rabbit IgG (Santa Cruz Biotechnology) or purified anti-NS1-BP antibody at 4°C for 2 h. Beads were washed with lysis buffer 4×, mixed with sample buffer and loaded onto a SDS-PAGE. After electrophoresis, the gel was stained with Colloidal blue (Invitrogen). Each lane was excised into 8 fragments containing proteins ranging from 250 kDa to 30 kDa. Gel slices were subjected to in-gel digestions followed by LC/MS/MS analysis that were performed at the Protein Identification core facility at University of Texas Southwestern Medical Center at Dallas. Acquired results were searched against the NCBI-nr protein database with Mascot software (Matrix Science).

### Size exclusion chromatography

A549 cells were grown to 90–100% confluency and mock-infected or infected with virus at MOI 5 for 5 h. Cells were lysed, as described in the previous section, and passed through QIAshredder columns (Qiagen). Lysates (8 mg) were centrifuged twice at 15000×g for 20 min and subjected to a Superdex 200 column (GE Healthcare). One-milliliter fractions were collected from number 1 to 25, and 200 µL of each fraction were precipitated with 2% trichloroacetic acid with 8 volume of cold acetone. The solution was incubated at −20°C overnight, and protein pellets were precipitated at 15000×g for 20 min and washed with cold acetone. The pellets were air dried, resuspended in sample buffer, and resolved by SDS-PAGE.

### RNA interference and transfection

siRNA oligos (siGENOME SMARTpool) designed for silencing human IVNS1ABP and hnRNPs were purchased from Dharmacon (Thermo Fisher Scientific). Non-targeting siRNAs, AUGAACGUGAAUUGCUCAAdTdT together with siGENOME non-targeting siRNA #3 (Thermo Fisher Scientific) were used as controls. The siRNAs were transfected with RNAiMAX (Invitrogen), 1.5 µL of RNAiMax was used per 25 pmol siRNA, according to manufacture's instruction. A549 cells were seeded into 12-well plates at a density 1×10^5^ the day before transfection, and cells were transfected with 50 nM of non-targeting siRNAs or siRNAs specific for NS1-BP or hnRNPs. Knockdown efficiency was detected by western blot 48 h after transfection.

### Isolation of RNAs associated with NS1-BP

RNA associated with NS1-BP were isolated as described [48]. In brief, A549 cells were grown to 90–100% confluency (∼7.5×10^6^ cells) and infected with influenza virus at MOI 5 for 5 h. To induce crosslinking, cells were incubated with 0.3% formaldehyde/PBS (Electron Microscopy Sciences) at room temperature for 10 min. Non-crosslink control was incubated with 10 mL PBS. Crosslinking reaction was quenched by adding 1.25 mL of 2 M glycine (pH 7.0) and incubated at room temperature for 5 min. Cells were lysed with RIPA buffer and then centrifuged at 16,000 g for 10 min at 4°C. Five percent of lysates were saved as input. Immunoprecipitation was performed as previously described with 6 µg of anti-NS1-BP antibodies or purified IgG from pre-immune rabbit serum. After washes, 100% beads and the 5% of input were mixed with reverse buffer [10 mM Tris (pH 6.8), 5 mM EDTA, 10 mM DTT, and 1% SDS] and incubated at 70°C for 45 min to reverse the cross-link. Samples were then mixed with Proteinase K at 37°C for 30 min, and RNA were extracted with Phenol∶Chloroform∶Isoamyl Alcohol (Invitrogen). After precipitation, RNA was subjected to DNase I digestion followed by phenol∶chloroform extraction. RNA was reversed transcribed into cDNA and analyzed by qPCR. The amount of RNA was calculated as 2^−Ct^, and the RNA associated with NS1-BP was normalized to the input amount and expressed as percentage of IP [%IP = (2^−Ct Bead^/2^−Ct input^)×5%]. To show the relative amount of different RNAs, we set the %IP of pull-down M segment to 1, and the %IP of each mRNA was normalized to the M segment.

### RNA purification and RT-qPCR

Total RNA was isolated from A549 cells with RNA Isolation Kit (Roche) and 0.5 µg of total RNA was reverse transcribed into cDNA by SuperScript II reverse transcriptase (Invitrogen). Reactions were set up according to the manufacturer's protocol in the presence of 1 µM of specific primers as following: oligo-(dT)_15_ for mRNA and random hexamers (Roche) for host 18S rRNA. RT was carried on 42°C for 1 h and inactivated by heating at 80°C for 20 min. RT reactions were then diluted with water at a ratio 1∶5, and 1.25 µL of cDNA mixture was subjected to quantitative real-time PCR (qPCR) using SYBR green I Master combined LightCycler 480 System (Roche). Real-time PCR was carried out by initial denaturation at 95°C for 3 min, 40 cycles of 95°C for 15 sec, 60°C for 15 sec, 72°C for 20 s, and followed by a melting curve cycle from 65°C to 95°C for quality assurance. Gene-specific primers were designed by D-LUX program (Invitrogen) and validated by analysis of template titration and dissociation curves. Primer Sequences used in this study are listed as follows (5′→3′):

18S rRNA (Forward: ACCGCAGCTAGGAATAATGGA, Reverse: GCCTCAGTTCCGAA AACCA); RPL32 (Forward: CGGCGTGCAACAAATCTTACTGTGCCG, Reverse: CCAG TTGGGCAGCTCTTTCC); GAPDH (Forward: CGACCGGAGTCAACGGATTTGGTCG, Reverse: CCAGTTGGGCAGCTCTTTCC); β-actin (Forward: CCGCGAGAAGATGACCC AGAT, Reverse: CGTTGGCACAGCCTGGATAGCAACG); α-tubulin (Forward: CACTCT GATTGTGCCTTCATGG, Reverse: CGAGCTTAGTGTAGGTTGGGCGCTCG); NS1-BP (Forward: CGCTGGTAATCAACTGGGTGCAGCG, Reverse: ACCTCTTCCATCAGCTC TTCCA); NS (Forward: CAGGACATACTGATGAGGATG, Reverse: GTTTCAGAGACTCGAACTGTG) NS1 (Forward: TGGAAAGCAAATA GTGGAGCG, Reverse: GTAGCGCGATGCAGGTACAGAG); NS2 (Forward: CAAGCTT TCAGGACATACTGATGAG, Reverse: CTTCTCCAAGCGAATCTCTGTAGA); M1 (Forward: ATCAGACATGAGAACAGAATGG, Reverse: TGCCTGGCCTGACTAGCAA TATC); M2 (Forward: CGAGGTCGAAACGCCTATCAGAAAC, Reverse: CCAATGATA TTTGCTGCAATGACGAG); mRNA_3_ (Forward: GCAAAAGCAGGGCCTATCAGAAAC, Reverse: CCAATGATA TTTGCTGCAATGACGAG); M4 (Forward: ACCGATCTTGAGGCCTATCAGAAAC, Reverse: CCAATGATA TTTGCTGCAATGACGAG).

### UV crosslinking and RNA-protein interaction

The full length M1 RNA (1070 nt) was transcribed from a PCR template by T7 polymerase using the standard GpppG cap analogue to cap the 5′ end and the indicated ^32^P-NTPs to ^32^P-labeled the RNA throughout it's length. Labeled M1 RNA (10 nM) was incubated with 60% JSL1 nuclear extract in a total volume of 12.5 µl under splicing condition, which contains (final concentration): 12 mM Tris-HCl, pH 7.5, 3.2 mM MgCl2, 1 mM ATP, 20 mM CP, 1 mM DTT, 0.125 U RNasin (Promega), 60 mM KCl and 12% glycerol. Reactions were incubated at 30°C for 20 min, crosslinked using UV light (254 nm) for 20 min on ice, and digested with RNaseT1 and RNase A for 20 min at 37°C. Reactions were resuspended in 2× SDS loading buffer, denatured for 5 min at 95°C, analyzed under denaturing conditions on an SDS-PAGE gel (Acrylamide/Bis 37.5∶1, BioRad), and detected by autoradiography. Immunoprecipitation after crosslinking was carried out as described previously [35], [36], with the antibodies against hnRNP L (4D11, abcam), hnRNP K (3C2, abcam), hnRNP A1 (4B10, abcam) hnRNP M (2A6, abcam), hnRNP U (3G6, abcam), and NS1BP.

### Plaque assay

One day before the assay, MDCK cells were seeded into 6-well plates at a density of 5×10^5^ cells/well. Ten-fold serial dilutions of each supernatant (from 10^−1^ to 10^−7^) were prepared in 400 µL PBS containing 100 units/mL penicillin, 100 µg/mL streptomycin, 0.2% BSA, 0.9 mM CaCl_2_, and 1.05 mM MgCl_2_. Cells were infected with 300 µL of each dilution. After 1 h of adsorption, inoculums were removed, and cells were overlaid with 2 mL of agar mixture [1× EMEM, 0.6% agar 100 units/mL penicillin, 100 µg/mL streptomycin, 2 mM L-glutamine, 0.2% bovine serum albumin (BSA), 10 mM HEPES, 0.22% sodium bicarbonate, 0.01% DEAE-Dextran, and 0.5 µg/mL TPCK-treated trypsin]. Plates were incubated at 37°C with 5% CO_2_ for 48 h. After 2 days, cells were fixed by adding 2 mL of 2% formaldehyde on the top of the agar and incubated at room temperature for 1 h. After removal of the agar, monolayers were stained with 0.1% crystal violet prepared in 0.25% methanol, and the plaques were counted.

### SDS-PAGE and western blot analysis

Gel electrophoresis was performed using the Tricine-SDS PAGE system as described [49]. Acrylamide gels used in this study were 7%, acrylamide solution was 40%, and acrylamide/bis-acrylamide ratio was 5%. Running gel buffer contained 1.5 M Tris-HCl, pH 8.45; stacking gel buffer contained 0.3 M Tris-HCl and 0.4% SDS, pH 7.8. Cathode buffer contained 0.1 M Tris, 0.1 M Tricine, and 0.1% SDS, pH 8.25. Anode buffer was 0.2 M Tris-HCl, pH 8.9. Thirty ml of the running gel mixture was prepared for 4 gels, which includes 5.25 mL of acrylamide solution, 10 mL of running gel buffer, 4 g glycerol, 10.64 mL water, 100 µL ammonium persulfate (10%), and 10 µL of TEMED (N,N,N′,N′-tetramethylethane-1,2-diamine). The stacking gel mixture contained 1.23 mL of acrylamide solution, 3 mL of stacking gel buffer, 7.77 mL of water, 100 µL of ammonium persulfate (10%), and 10 µL of TEMED. Samples were mixed with equal volume of 2× sample buffer [100 mM Tris-HCl, 8% SDS, 24% glycerol, 200 mM DTT, and 0.02% bromophenol blue, pH 7.8], heated at 95°C for 10 min, and loaded onto the wells. The electrophoresis was performed at a constant voltage of 120 V until the tracking dye reached the edge of the gel.

After electrophoresis, proteins were transferred to a PVDF membrane (Millipore) using a mini transfer electrophoretic cell with transfer buffer [20 mM Tris, 192 mM Glycine, and 10% methanol] at a constant voltage of 30 V overnight. The PVDF membrane was then blocked with 5% skim milk in PBS for 1 h, followed by two PBS-T (0.05% Tween 20 in PBS) washes, and incubated with primary antibody (prepared in 10 mg/mL BSA/PBS) at room temperature for 1 h. After incubation, the membrane was washed 5 times with PBS-T buffer for 5 min each, and then incubated with HRP-conjugated secondary antibodies for another hour. Finally, the PVDF membrane was washed 4 times with PBS-T buffer for 10 min each, submerged in ECL reagent (34096, Pierce, Thermo Scientific) for 5 min, and exposed to a film in the dark room for 20 s to 5 min until the signal was visible.

### Expression and purification of GST-NS1-BP

The plasmid pGEX-NS1BP was transformed into BL21(DE3) cells. A single colony was inoculated into 2.5 ml of LB with 100 µg/ml of ampicillin and incubated at 37°C in a shaker at 225 rpm overnight. Next day, the overnight culture was added into 250 ml of 2-YT broth (Invitrogen) and grown to OD 0.6 at 37°C. Protein expression was induced by 0.2 mM isopropyl-beta-D-thiogalactopyranoside (IPTG) at 20°C for 6 h. Bacteria were pelleted and re-suspended in 25 ml of cold lysis buffer [20 mM HEPES, pH 7.5, 1 mM EDTA, 300 mM NaCl, and 1× protease inhibitor cocktail (Roche)]. Cells were lysed by five passages through an EmulsiFlex-C5 homogenizer (Avestin, Canada) at 8000 psi, and lysates were centrifuged at 13,000×g for 15 min. The supernatant was incubated with 2 ml (50% slurry) glutathione Sepharose beads at 4°C for 1 h, and beads were washed 10 times with cold lysis buffer. GST-NS1-BP was eluted with 50 mM glutathione solution (pH 7.5).

### Immunostaining and fluorescence microscopy

A549 cells were seeded in 12-well plates at a density of 1×10^5^ cells/well the day before siRNA transfection. At the second day, cells were treated with 50 nM of non-targeting or NS1-BP siRNAs. After 24 h, cells in each well were trypsinized and split into 4 wells of 24-well plate including a coverslip per well, and incubated for another 24 hours. To synchronize the infection, cells were pre-chilled on ice for 10 min before infection, washed with cold PBS, and infected with influenza virus at MOI 50. After 45 min adsorption on ice, cells were washed with PBS and overlaid with 0.5 ml of warm infection media including 1 mM cycloheximide. At the indicated time points, cells on coverslips were fixed with 3% formaldehyde/PBS at room temperature for 15 min, washed 3× with PBS, and then permeabilized with 0.5% TritonX-100/PBS at room temperature for 5 min. Then, coverslips were incubated with NP antibody (ab20343, Abcam) prepared in BSA solution (1 mg/ml) at a dilution of 1∶3200 at room temperature for 1 h. After washed 3× in PBS, coverslips were incubated with Alexa Fluor 488 donkey anti-mouse IgG (Invitrogen) at a dilution of 1∶500 in BSA for another 1 h, followed by three washes in PBS. Coverslips were further incubated with Hoechst (0.5 µg/ml) (Invitrogen) at room temperature for 5 min and washed in PBS. After the final PBS wash, coverslips were mounted onto glass microscope slides with mounting media (Dako, Denmark). Images were taken and processed in the Zeiss Axioplan 2E microscope, Hamamatsu monochrome digital camera, and OpenLab software.

### Accession numbers

NS1-BP (NM_006469); hnRNP M (EAW68921) hnRNP A1 (P09651); hnRNP U (Q00839); DDX17 (CAG30318); Hsp 70 (NP_006588); Hsp90 (AAA36025); NFAR-2 (AAD51099); PARP-1 (EAW69785); polyadenylate-binding protein 4 (NP_003810); Pol II CTD (NM_000937), Influenza virus (A/WSN/1933(H1N1)) strain: PB1 (CY034138); PA (CY034137); M1 (L25818); NP (ACF54602); M2 (AAA91324); NS1 (K01076); NS2 (ABF47961).

## Supporting Information

Figure S1 **NS1-BP depletion does not affect virus entry and vRNP nuclear import.** A549 cells were grown on coverslips, transfected with non-targeting or NS1-BP siRNAs for 48 h, and infected with A/WSN/33 at MOI 50 on ice to synchronize infection. After virus adsorption, cells were incubated with warm media containing 1 mM cycloheximide and cells were then fixed with 3% formaldehyde at each indicated time point. (A) Cells were stained with Hoechst (top panels) and antibody against influenza A virus nucleoprotein (NP) (bottom panels). Scale bar: 10 µm. (B) The ratio of NP nuclear localization/cell number at 3.5 h post-infection was determined in control and NS1-BP-depleted cells. The results represent the average ratio from three independent experiments and ∼150 cells were counted in each experiment. Error bars are mean±SD (n = 3). (C) and (D) Control for cycloheximide treatment showing low levels of p53, which is short-lived. In parallel wells, cells were transfected with non-targeting or NS1-BP siRNAs for 48 h and used as a control to demonstrate knockdown efficiency.
(TIFF)

Figure S2 **hnRNP L but not hnRNP K bind to CD45 exon 4.** A 100 nt, capped and ^32^P-labeled RNA corresponding to exon 4 of the human CD45 gene was incubated in nuclear extract and crosslinked with UV light to analyze protein-RNA complexes. Antibodies specific to hnRNP L efficiently immunoprecipitated a protein-RNA complex while no bound protein was observed with antibody to hnRNP K or an IgG control. Molecular weight markers and a total crosslink reaction are shown.
(TIFF)

Figure S3 **hnRNPs A1, L, and M do not alter M2 expression.** A549 cells were transfected with non-targeting or hnRNPA A1, L, and M siRNAs for 48 h prior to infection. siRNA transfected cells were infected with A/WSN/33 at MOI 2. Cells were harvested at the indicated hours post-infection, and viral protein accumulation was assessed by immunoblot analysis. Each protein band was quantified by ImageJ and normalized to α-tubulin levels.
(TIFF)

Figure S4 **Detection of NS1-BP and hnRNP U in JSL1 nuclear extract.** JSL1 nuclear extract was subjected to immunoblot analysis with antibodies specific to hnRNP U (A) and NS1-BP (B), detected using different amounts of extract. MW, molecular weight markers.
(TIFF)

Table S1 **Identification of NS1-BP binding proteins by mass spectrometry.** HeLa cell lysates were immunoprecipitated with control IgG or NS1-BP antibodies in the absence of RNase. Interacting proteins were resolved by SDS-PAGE and identified by mass spectrometry. The gel lane was divided into eight segments from molecular weight 250 kDa to 35 kDa, and the top hits of each segment are listed in the table.
(DOCX)
