# Supplementary figures and images for: Cellular RNA Binding Proteins NS1-BP and hnRNP K Regulate Influenza A Virus RNA Splicing
Source: PLoS Pathog. 2013 Jun 27;9(6):e1003460. doi: 10.1371/journal.ppat.1003460 (PMC3694860; doi:10.1371/journal.ppat.1003460)

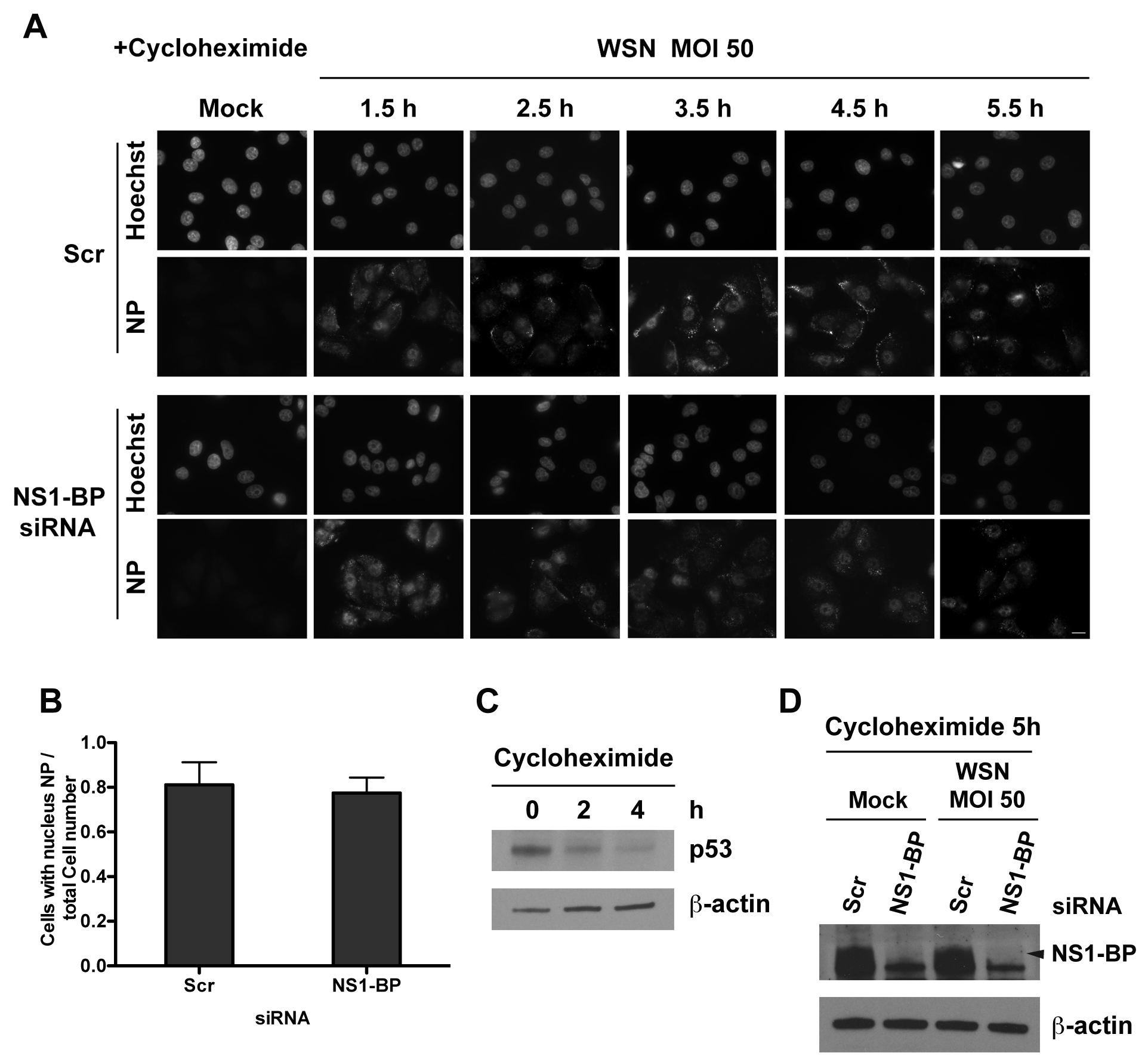

Supplement: Figure S1 — NS1-BP depletion does not affect virus entry and vRNP nuclear import. A549 cells were grown on coverslips, transfected with non-targeting or NS1-BP siRNAs for 48 h, and infected with A/WSN/33 at MOI 50 on ice to synchronize infection. After virus adsorption, cells were incubated with warm media containing 1 mM cycloheximide and cells were then fixed with 3% formaldehyde at each indicated time point. (A) Cells were stained with Hoechst (top panels) and antibody against influenza A virus nucleoprotein (NP) (bottom panels). Scale bar: 10 µm. (B) The ratio of NP nuclear localization/cell number at 3.5 h post-infection was determined in control and NS1-BP-depleted cells. The results represent the average ratio from three independent experiments and ∼150 cells were counted in each experiment. Error bars are mean±SD (n = 3). (C) and (D) Control for cycloheximide treatment showing low levels of p53, which is short-lived. In parallel wells, cells were transfected with non-targeting or NS1-BP siRNAs for 48 h and used as a control to demonstrate knockdown efficiency. (TIFF) [file ppat.1003460.s001.tiff]

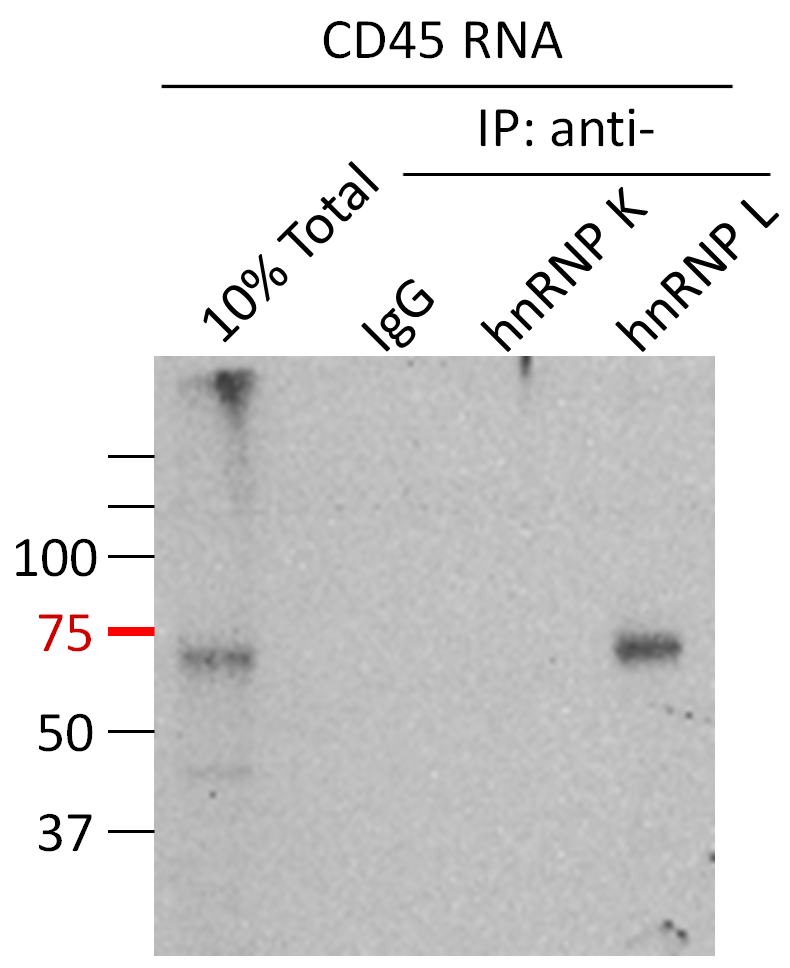

Supplement: Figure S2 — hnRNP L but not hnRNP K bind to CD45 exon 4. A 100 nt, capped and 32P-labeled RNA corresponding to exon 4 of the human CD45 gene was incubated in nuclear extract and crosslinked with UV light to analyze protein-RNA complexes. Antibodies specific to hnRNP L efficiently immunoprecipitated a protein-RNA complex while no bound protein was observed with antibody to hnRNP K or an IgG control. Molecular weight markers and a total crosslink reaction are shown. (TIFF) [file ppat.1003460.s002.tiff]

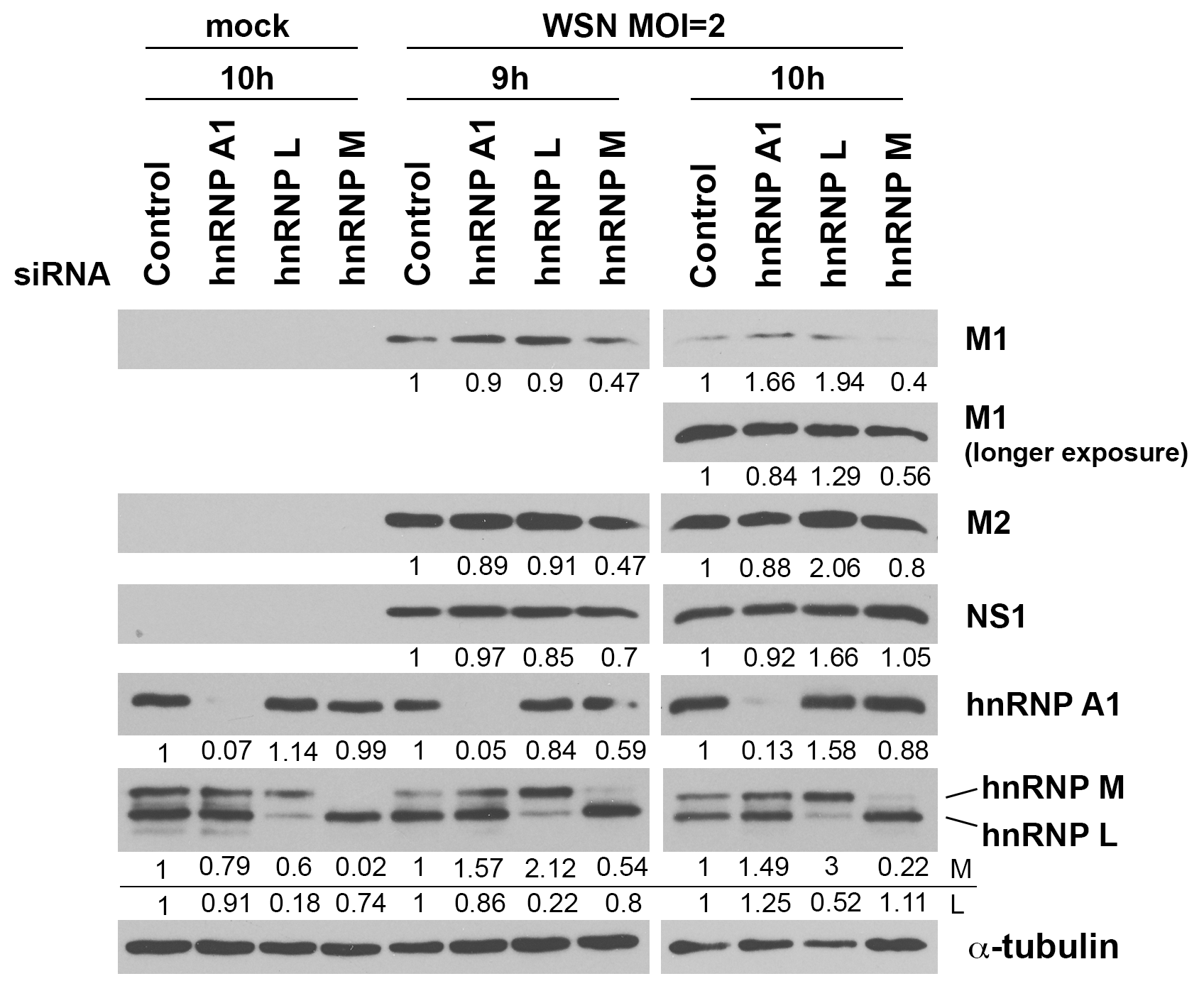

Supplement: Figure S3 — hnRNPs A1, L, and M do not alter M2 expression. A549 cells were transfected with non-targeting or hnRNPA A1, L, and M siRNAs for 48 h prior to infection. siRNA transfected cells were infected with A/WSN/33 at MOI 2. Cells were harvested at the indicated hours post-infection, and viral protein accumulation was assessed by immunoblot analysis. Each protein band was quantified by ImageJ and normalized to α-tubulin levels. (TIFF) [file ppat.1003460.s003.tiff]

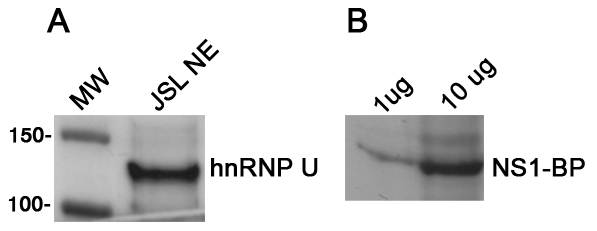

Supplement: Figure S4 — Detection of NS1-BP and hnRNP U in JSL1 nuclear extract. JSL1 nuclear extract was subjected to immunoblot analysis with antibodies specific to hnRNP U (A) and NS1-BP (B), detected using different amounts of extract. MW, molecular weight markers. (TIFF) [file ppat.1003460.s004.tiff]
