# Supplementary material for: Cellular RNA Binding Proteins NS1-BP and hnRNP K Regulate Influenza A Virus RNA Splicing
Source: PLoS Pathog. 2013 Jun 27;9(6):e1003460. doi: 10.1371/journal.ppat.1003460 (PMC3694860; doi:10.1371/journal.ppat.1003460)
Supplement: Table S1 — Identification of NS1-BP binding proteins by mass spectrometry. HeLa cell lysates were immunoprecipitated with control IgG or NS1-BP antibodies in the absence of RNase. Interacting proteins were resolved by SDS-PAGE and identified by mass spectrometry. The gel lane was divided into eight segments from molecular weight 250 kDa to 35 kDa, and the top hits of each segment are listed in the table. (DOCX) [file ppat.1003460.s005.docx]

**Table S1. NS1-BP binding partners**

| **Band (kDa)** | **Accession No.** | **Protein Name** | **Score** | **Theoretical MW (kDa)** | **No. Masses Matched** | **Sequence Coverage%** |
| --- | --- | --- | --- | --- | --- | --- |
| #1  170-220 | gi\|4758416  gi\|12667788 | Golgi-specific brefeldin A resistance factor 1  Myosin, heavy polypeptide 9, non-muscle | 1255  246 | 206.3  226.4 | 101  39 | 23.6  14.3 |
| #2  130-170 | gi\|17380291  gi\|31621305  gi\|100913206  gi\|119582037  gi\|4827040  gi\|20336290 | Regulator of nonsense transcripts 1  Leucine-rich PPR motif-containing protein  DEAH (Asp-Glu-Ala-His) box polypeptide 9  La ribonucleoprotein domain family, member 1  Thyroid hormone receptor associated protein 3  DEAH (Asp-Glu-Ala-His) box polypeptide 30, isoform 2 | 471  421  373  203  187  173 | 124.3  157.8  140.9  129.2  108.6  129.4 | 40  39  57  35  15  19 | 29.2  27.4  25.7  26.5  14.4  13.5 |
| #3  120-130 | gi\|74136883  gi\|5762315  gi\|119599167  gi\|119590191 | Heterogeneous nuclear ribonucleoprotein U isoform a  Nuclear factor associated with dsRNA NFAR-2  DEAH (Asp-Glu-Ala-His) box polypeptide 36, isoform CRA_a  Poly (ADP-ribose) polymerase | 884  224  210  188 | 90.5  95.3  114.7  113.0 | 140  19  17  12 | 31.8  18.1  14.8  13.5 |
| #4  95-120 | gi\|38201710  gi\|27477136  gi\|306891  gi\|4505343  gi\|50659095  gi\|3319956 | DEAD box polypeptide 17 isoform 1  Zinc finger antiviral protein isoform 1  90kDa heat shock protein  Nuclear cap binding protein subunit 1, 80kDa  DEAD (Asp-Glu-Ala-Asp) box polypeptide 21  E1B-55kDa-associated protein | 309  216  166  161  159  99 | 80.2  101.4  83.2  91.8  87.3  95.8 | 30  9  19  16  28  14 | 26.5  12.2  17.1  14.7  26.9  16.7 |
| #5  72-95  **Band (kDa)** | gi\|4504715  gi\|56237027  gi\|5729877  gi\|119589327  gi\|24475847  gi\|5031755  **Accession No.** | Poly(A) binding protein, cytoplasmic 4  Insulin-like growth factor 2 mRNA binding protein 1  heat shock 70kDa protein 8 isoform 1  Heterogeneous nuclear ribonucleoprotein M, isoform CRA_c  Influenza virus NS1A binding protein (NS1-BP)  Heterogeneous nuclear ribonucleoprotein R isoform 2  **Protein Name** | 607  495  474  398  209  165  **Score** | 70.7  63.4  70.9  77.6  71.7  70.9  **Theoretical MW (kDa)** | 73  67  49  72  53  25  **No. Masses Matched** | 43.6  44.9  38.5  40.8  24.8  24.5  **Sequence Coverage%** |
| #6  55-72 | gi\|11527777  gi\|14165437  gi\|5805295  gi\|4335947 | Heterogeneous nuclear ribonucleoprotein L  Heterogeneous nuclear ribonucleoprotein K, isoform a  RNA-binding protein isoform G3BP-2a  Staufen protein | 493  469  159  141 | 60.1  51.0  54.1  55.0 | 74  82  26  11 | 42.4  44.4  31.1  23 |
| #7  45-50 | gi\|181486  gi\|14110417  gi\|532313  gi\|14249959  gi\|4826760  gi\|4503571  gi\|3256007 | DNA-binding protein B  Heterogeneous nuclear ribonucleoprotein D, isoform b  NF45 protein  Heterogeneous nuclear ribonucleoprotein C (C1/C2)  Heterogeneous nuclear ribonucleoprotein F  Enolase 1  Heterogeneous nuclear ribonucleoprotein G | 644  563  366  331  251  234  219 | 40.0  36.2  44.7  32.4  45.6  47.1  42.4 | 43  68  34  15  13  13  36 | 49.2  37.8  36.5  34.1  25.8  27.2  43.7 |
| #8  40-45 | gi\|55956921  gi\|38201714  gi\|4501885 | Heterogeneous nuclear ribonucleoprotein AB isoform b  ELAV-like 1  beta actin | NA  NA  NA | 30.6  36.1  41.7 | NA  NA  NA | NA  NA  NA |
